# Retrospective analysis of severe COVID-19 pneumonia patients treated with lopinavir/ritonavir: A comparison with survivor and non-survivor patients

**DOI:** 10.4102/sajid.v35i1.233

**Published:** 2020-12-21

**Authors:** Omer Ayten, Bengu Saylan

**Affiliations:** 1 Department of Pulmonology Medicine, Faculty of Health Sciences, Sultan Abdulhamit Han Education and Training Hospital, Uskudar/Istanbul, Turkey

**Keywords:** COVID-19, lopinavir, ritonavir, mortality, severe pneumonia

## Abstract

**Background:**

Currently there is no proven medical treatment for COVID-19. We aimed to determine the factors affecting mortality and changes in clinical and laboratory findings in patients with severe COVID-19 pneumonia treated with lopinavir/ritonavir (Lpv/r).

**Methods:**

Data of survivor and non-survivor severe COVID-19 pneumonia patients treated with Lpv/r were analysed retrospectively.

**Results:**

A total of 73 patients, 26 (35.6%) females and 47 (64.4%) males were included in the study. The mean age of non-survivor and survivor patients was 64.3 ± 12 and 52.6 ± 12.2, respectively (*p* < 0.0001). Frequency of smoking and comorbid diseases was higher in non-survivor patients than in survivor patients (37% vs. 8.7% *p* = 0.003 and 92.6% vs. 28.3%, *p* < 0.0001, respectively). Age (Odds ratio [OR] 1.09, 95% confidence interval [95% CI] 1.03–1.14), smoking (OR 6.18, 1.7–22.42), presence of comorbid disease (OR 31.73, 6.26–153.56), coronary artery disease (OR 9.26, 1.79–47.77), arrhythmia (OR 13.8, 1.56–122.22), hypertension (OR 14, 4.28–45.74), diabetes (OR 7.22, 2–25.99) and congestive heart failure (OR 10.22, 1.13–92.93) were statistically associated with increased mortality (*p* < 0.05). Also increased neutrophil (OR 1.26, 1.08–1.46), C-reactive protein (CRP) (OR 1.01, 1.003–0.02), lactate dehydrogenase (LDH), (OR 1.002, 1.001–1.004), D-dimer (OR 1.001, 1.002–1.003), and aspartate transaminase (AST) levels (OR 1.05, 1.02–1.08 were associated with increased mortality.

**Conclusion:**

The presence of advanced age, active smoking, comorbidity, especially hypertension, diabetes, arrhythmia, coronary artery disease, congestive heart failure and neutrophil, C-reactive protein, lactate dehydrogenase, D-dimer and aspartate transaminase were associated with mortality. The efficacy of Lpv/r, warrants further verification in future studies.

## Introduction

On December 2019, pneumonia cases caused by a new beta coronavirus turned into a pandemic that affected the whole world, starting in Wuhan, China. The World Health Organization (WHO) named this virus as severe acute respiratory syndrome coronavirus 2 (SARS-CoV-2), and the disease caused by the virus was named as coronavirus disease-19 (COVID-19). By the end of May 2020, the number of people infected with SARS-CoV-2 reached 6 million worldwide, and the number of COVID-19 related deaths exceeded 360 000. In the same period, 171 000 people were infected in Turkey and 4700 of them died because of COVID-19.^1^

Currently, there is no proven medical treatment for COVID-19. Unfortunately, treatment is planned experimentally by taking into account the clinical experiences of the 2003 SARS and 2012 MERS outbreaks and the antiviral efficacy of some drugs. However, there are not enough studies on the effectiveness of the antiviral drugs and treatment methods to be used at different stages of the disease. Therefore, treatment guidelines and used antiviral agents differ amongst countries.^2^ Lopinavir/ritonavir (Lpv/r) are proteinase inhibitors consisting of a combination of lopinavir and ritonavir. Lopinavir/ritonavir is thought to act by inhibiting 3CLpro proteinase, which is responsible for the processing of polypeptide product into protein components in the RNA genome in coronaviruses.^3^ Scientific Committee of Turkish Ministry of Health guidelines of COVID-19 recommends Lpv/r use only in hypoxic patients with severe pneumonia (SpO_2_ level in the room air < 90%) and patients with an intermediate level disease in the presence of signs of progression under treatment with hydroxychloroquine.^4^

The use of Lpv/r as an initial treatment method in SARS patients has been shown to inhibit SARS-CoV activation within 48 h at serum concentrations of 4 µ/mL and 50 mg/mL (2.4% and 28.8%, respectively).^5^ It has been shown that the use of Lpv/r as an initial treatment in severe acute respiratory failure because of SARS significantly reduces mortality (15.6% and 2.3%, respectively), intubation requirement (11% and 0%, respectively) and required doses of steroid.^6^

The positive effect of using Lpv/r as an initial treatment in SARS patients has not yet been demonstrated in COVID-19 patients. In a limited number of studies, it has been shown that the use of Lpv/r in COVID-19 patients improves symptoms, reduces radiological recovery time and nasopharyngeal cleaning time.^7,8^ In a new published study, Lpv/r treatment added to standard supportive care was not associated with clinical improvement or mortality in seriously ill patients with COVID-19 different from that associated with standard care alone.^9^

In this study, we aimed to determine the factors affecting mortality in patients with COVID-19-induced pneumonia using Lpv/r and the changes in clinical and laboratory findings with Lpv/r treatment. We compared patients who died and survived under Lpv/r treatment in terms of demographic characteristics, smoking, comorbidities, laboratory parameters and clinical outcomes.

## Materials and methods

### Study design, population

This was a retrospective observational study conducted between 10 March 2020 and 15 April 2020 at Istanbul Sultan Abdulhamid Han Education and Research Hospital. A total of 73 severe or critical COVID-19 patients treated with Lpv/r, were included in the study. For all patients pneumonia was confirmed by chest computed tomography (CT). Severe illness was defined as patients with respiratory rate > 30/min and SpO_2_ level in the room air < 90%. Critical illness was defined as patients with respiratory failure, septic shock or multiple organ failure treated in the intensive care unit (ICU).

Inclusion criteria for the study were as follows:

1. patients who were 18 years of age or older and have showed a positive COVID-19 PCR
2. for COVID-19 PCR negative patients, COVİD-19 diagnosis conducted with clinical, laboratory and radiological findings
3. to have new emerging infiltration compatible with COVID-19 pneumonia in CT
4. SpO_2_ level in the room air < 90%
5. Lpv/r treatment for at least 7 days.

Exclusion criteria for the study were as follows:

1. patients under the age of 18
2. patients who were pregnant or breastfeeding a baby
3. patients who had no findings compatible with COVID-19 pneumonia in CT
4. SpO_2_ level in the room air ≥ 90
5. Lpv/r treatment for less than 7 days.

Severe acute respiratory syndrome coronavirus 2 diagnosis was performed by using next-generation sequencing or RT-PCR testing. The patients were divided into two groups comprising non-survivors and survivors. Both groups were compared in terms of clinical features, laboratory findings and factors affecting mortality.

### Lopinavir or ritonavir treatment protocol

All patients used Lpv/r in a treatment scheme according to the national guidelines. Lopinavir/ritonavir was used only in hypoxic patients with severe pneumonia (SpO2 level in the room air < 90%) and patients with an intermediate level disease in the presence of signs of progression under treatment with hydroxychloroquine. Lopinavir/ritonavir treatment was started when the room air SO2 was < 90 and it was used for at least 7 days twice a day with a 200/50 mg dosage.

### Collection of data and evaluation of treatment effectiveness

The demographic and clinical features, laboratory findings and treatment characteristics of the patients were reviewed using hospital electronic medical records system. Demographic features, symptoms and duration of symptoms, comorbidities, laboratory parameters before and after treatment including white blood cell count (WBC), neutrophil, lymphocyte, platelet, alanine aminotransferase (ALT), aspartate transaminase (AST), lactate dehydrogenase (LDH), total bilirubin, D-dimer, C-reactive protein (CRP) were recorded. The starting time (day of hospitalisation) and duration of Lpv/r treatment were noted. Additionally, symptom duration until hospitalisation, treatment location (intensive care/clinic), total hospitalisation time (clinical or intensive care) and additional therapies (vitamin C) and PCR negativity time were recorded. Smoking status was classified as active smoker or not active smoker. Computed tomography was performed on all patients. The severity of the radiological findings was evaluated by determining scores between 0 and 20 according to the degree of involvement of each lung lobe, similar to the criteria set by Chang and colleagues.^10^ According to involvement of the disease, each of the five lobes was scored as:

- 0 point (0%)
- 1 points minimal (1%–25%)
- 2 points mild (26%–50%)
- 3 points moderate (51%–75%)
- 4 points severe (76%–100%)

Total radiological weight score was obtained by summing the scores of five lobes (0–20).

### Statistical analysis of data

As a result of the suitability of the Central Limit Theorem, parametric tests were used without normality test.^11^ Non-parametric tests were used in laboratory parameters with high deviations from the mean. While performing statistic data of continuous structure, average, and standard deviation, median and 25% – 75% quarter values were used. When defining categorical variables, frequency and percentage values were used. Student’s *t*/Mann–Whitney *U* test statistics were used to compare the mean of the two groups. Paired-t/Wilcoxon test was used to compare the averages of the two dependent groups. Chi-square test statistics were used to evaluate the relationship between categorical variables. Exposure ratios (odds ratio [OR]) of variables that are considered to be related to mortality are given.

As the Life Analysis, the test statistic ‘Log-Rank’ Test was used in the Kaplan–Meier method. The statistical significance level of the data was taken as *p* < 0.05. MedCalc statistics package program and www.e-picos.com New York, New York software were used to evaluate the data.

### Ethical consideration

Ethical approval of the study was obtained from Umraniye Training and Research Hospital Ethics Committee dated 12 May 2020 and numbered 161.

## Results

A total of 73 patients, 26 females (35.6%) and 47 males (64.4%), were included in the study. Patient characteristics are summarised in Table 1. The mean age of the patients was 56.9 ± 13.3. The patients were divided into two groups comprising survivors (*n* = 46) and non-survivors (*n* = 27). The mortality rate was 36.9%. The average age of non-survivor patients (64.3 ± 12) was higher than survivor patients (52.6 ± 12.2) (*p* < 0.0001). A total of 19.2% (*n* = 14) of the patients were smoking. The frequency of smoking in non-survivor patients (37%, *n* = 10) was higher compared to that in survivor patients (8.7%, *n* = 4) (*p* = 0.003). During the application, COVID-19 PCR test was positive in 72.6% (*n* = 53) of the patients. Survivor patients had higher PCR negativity at admission compared to non-survivor patients (37% vs. 11%, *p* = 0.02, respectively). A total of 40 patients were followed up in the clinic (54.8%), 20 patients in the clinic + intensive care (27.4%) and 13 patients in intensive care (13.8%; Table 1). At least one comorbid disease was present in 38 patients (52.1%). The frequency of comorbid diseases in non-survivor patients was higher than in survivor patients (92.6% vs. 28.3%, *p* < 0.0001). In non-survivor patients, hypertension (81.5% vs. 23.9%), diabetes (40.7% vs. 8.7%), coronary artery disease (29.6% vs. 4.3%), arrhythmia (23% vs. 0%) congestive heart failure (22.2% vs. 0%) and malignancy (11.1% vs. 0%) were more common (*p* < 0.05; Table 1). In the survivor group, the frequency of stage 1 involvement in chest CT (*n* = 9, 19.6%) was significantly higher than the non-survivor group (0%; *p* = 0.05). There was no statistically significant difference between survivor and non-survivor patients in terms of fever, cough, shortness of breath, muscle pain, headache, GIS symptoms, COVID-19 contact history and vitamin C use (*p* > 0.05; Table 1).

**TABLE 1:** Demographics, baseline and clinical characteristics at presentation of patients treated with lopinavir/ritonavir.

Variables	Total (*n* = 73)		Survivor (*n* = 46)		Non survivor (*n* = 27)		*p* *
	*n*	%	*n*	%	*n*	%	
**Gender**	**-**	**-**	**-**	**-**	**-**	**-**	**0.18**
Male	47	64.4	27	58.7	20	74.1	-
Female	26	35.6	19	41.3	7	25.9	-
**Age** †	**-**	**-**	**-**	**-**	**-**	**-**	**< 0.0001**
**Smoking**	**-**	**-**	**-**	**-**	**-**	**-**	**0.003**
Yes	14	19.2	4	8.7	10	37	-
No	59	80.8	42	91.3	17	63	-
**Covid-19 contact**	**-**	**-**	**-**	**-**	**-**	**-**	**0.59**
Yes	16	21.9	11	23.9	5	18.5	-
No	57	78.1	35	76.1	22	81.5	-
**Covid PCR +**	**-**	**-**	**-**	**-**	**-**	**-**	**0.02**
Yes	53	72.6	29	63	24	88.9	-
No	20	27.4	17	37	3	11.1	-
**Comorbidities**	**-**	**-**	**-**	**-**	**-**	**-**	**< 0.0001**
Yes	38	52.1	13	28.3	25	92.6	-
No	35	47.9	33	71.7	2	7.4	-
**Hypertension**	**-**	**-**	**-**	**-**	**-**	**-**	**< 0.0001**
Yes	33	45.2	11	23.9	22	81.5	-
No	40	54.8	35	76.1	5	18.5	-
**Diabetes**	**-**	**-**	**-**	**-**	**-**	**-**	**0.001**
Yes	15	20.5	4	8.7	11	40.7	-
No	58	79.5	42	91.3	16	59.3	-
**Arrhythmia**	**-**	**-**	**-**	**-**	**-**	**-**	**0.001**
Yes	6	8.3	-	-	6	23.1	-
No	66	91.7	46	100	20	76.9	-
**CAD**	**-**	**-**	**-**	**-**	**-**	**-**	**0.002**
Yes	10	13.7	2	4.3	8	29.6	**-**
No	63	86.3	44	95.7	19	70.4	**-**
**CHF**	**-**	**-**	**-**	**-**	**-**	**-**	**0.001**
Yes	6	8.2	-	-	6	22.2	-
No	67	91.8	46	100	21	77.8	-
**COPD**	**-**	**-**	**-**	**-**	**-**	**-**	**0.89**
Yes	3	4.1	2	4.3	1	3.7	-
No	70	95.9	44	95.7	26	96.3	-
**Asthma**	**-**	**-**	**-**	**-**	**-**	**-**	**0.19**
Yes	1	1.4	-	-	1	3.7	-
No	72	98.6	46	100	26	96.3	-
**Malignancy**	**-**	**-**	**-**	**-**	**-**	**-**	**0.02**
Yes	3	4.1	-	-	3	11.1	-
No	70	95.9	46	100	24	88.9	-
**Immunosuppression**	**-**	**-**	**-**	**-**	**-**	**-**	**0.06**
Yes	2	2.7	-	-	2	7.4	-
No	71	97.3	46	100	25	92.6	-
**Signs and symptoms**	**-**	**-**	**-**	**-**	**-**	**-**	**-**
**Fever**	**-**	**-**	**-**	**-**	**-**	**-**	**0.9**
Yes	48	65.8	30	65.2	18	66.7	-
No	25	34.2	16	34.8	9	33.3	-
**Cough**	**-**	**-**	**-**	**-**	**-**	**-**	**0.14**
Yes	38	52.1	27	58.7	11	40.7	-
No	35	47.9	19	41.3	16	59.6	-
**Dyspnea**	**-**	**-**	**-**	**-**	**-**	**-**	**0.45**
Yes	20	24.7	14	30.4	6	22.2	-
No	53	72.6	32	69.6	21	77.8	-
**GİS (diarrhea and nausea)**	**-**	**-**	**-**	**-**	**-**	**-**	**0.71**
Yes	12	16.4	7	15.2	5	18.5	-
No	61	83.6	39	84.8	22	81.5	-
**Myalgia**	**-**	**-**	**-**	**-**	**-**	**-**	**0.91**
Yes	14	19.2	9	19.6	5	18.5	-
No	59	80.8	37	80.4	22	81.5	-
**Headache**	**-**	**-**	**-**	**-**	**-**	**-**	**0.62**
Yes	9	12.3	5	10.9	4	14.8	-
No	64	87.7	41	89.1	23	85.2	-
**Treatment location**	**-**	**-**	**-**	**-**	**-**	**-**	**< 0.0001**
Clinic	40	54.8	40	87.0	-	-	-
Clinic + ICU	20	27.4	5	10.9	15	55.6	-
ICU	13	17.8	1	2.2	12	44.4	-
**Radiology**	**-**	**-**	**-**	**-**	**-**	**-**	**0.05**
1	9	12.3	9	19.6	-	-	-
2	36	49.3	23	50.0	13	48.1	-
3	23	31.5	11	23.9	12	44.4	-
4	5	6.8	3	6.5	2	7.4	-
**Vitamin C**	**-**	**-**	**-**	**-**	**-**	**-**	**0.79**
Yes	38	53.5	23	52.3	15	55.6	-
No	33	46.5	21	47.7	12	44.4	-

† , Mean (SD): Total, 56.9 ± 13.3 (26–89); Survivor, 52.6 ± 12.2 (26–79); Non survivor, 64.3 ± 12 (35–89); (Mean = years; SD = Min–Max).

*N*, number; SD, standard deviation; Min, minimum; Max, maximum; CAD, coronary artery disease; CHF, congestive heart failure; COPD, chronic obstructive pulmonary disease; ICU, intensive care unit.

* , *p* is significant at the level of < 0.05. (Chi-square test).

Laboratory findings before and after treatment of both groups are summarised in Table 2. Neutrophil count, CRP, AST, total bilirubin, LDH and D-dimer levels were statistically higher in the non-survivor group before the Lpv/r treatment when compared with the survivor group (*p* < 0.05). After treatment, neutrophil, CRP, AST, ALT, LDH and D-dimer levels were increased and lymphocyte levels were decreased in the non-survivor group when compared with the survivor group (*p* < 0.05). The duration of clinical stay (5.4 ± 3.6 days) in the non-survivor group was shorter than the survivor group (11.9 ± 4.2 days). There was no statistical difference between the two groups in terms of symptom duration until hospitalisation (day), total length of stay (day), Lpv/r starting time and COVID-19 PCR negativity time (day) (*p* > 0.05). In the non-survivor group, CRP, LDH, D-dimer, ALT, AST values increased after treatment and SpO_2_ decreased (*p* < 0.05; Table 3). In the survivor group, after Lpv/r treatment, a statistically significant increase in platelet, CRP, ALT and SpO_2_ values and a decrease in LDH were observed (*p* < 0.05; Table 4).

**TABLE 2:** Comparison of laboratory and treatment parameters patients treated with lopinavir/ritonavir.

Variables	Survivor (*n* = 46) x̄ ± SD	Non survivor (*n* = 27) x̄ ± SD	*p* *
**Pre-treatment laboratory parameters**			
Lymphocyte (K/mm^3^)	1.23 ± 0.57	1.13 ± 0.67	0.46*
Neutrophil (K/mm^3^)	4.83 ± 3.09	7.94 ± 4.26	0.001*
LDH (U/L)	511.6 ± 271.7	830.44 ± 480.17	0.001*
D-dimer (µg/L)	800.4 ± 906.33	5080.91 ± 8545.47	0.005**
CRP (mg/L)	52.11 ± 58.54	136.97 ± 16.44	0.001**
ALT (IU/L)	30.26 ± 13.2	36 ± 24.67	0.20*
AST (IU/L)	27.8 ± 13.94	43.15 ± 25.92	0.002*
Total bilirubin (mg/dL)	0.59 ± 0.29	0.8 ± 0.61	0.05*
Symptom duration until hospitalisation (day)	4.1 ± 2.2	4.3 ± 2.1	0.77
Clinical length of stay (day)	11.9 ± 4.2	5.4 ± 3.6	< 0.0001
Total length of stay (day)	14.3 ± 7	12.1 ± 9.8	0.27
COVİD-PCR negativity time (day)	17.6 ± 6.5	20 ± 2.8	0.62
Starting day of Lpv/r	3.9 ± 2.4 (1–9)	3.7 ± 3.1 (1–8)	0.73

*N*, number; SD, standard deviation; LDH, lactate dehydrogenase; CRP, C-reactive protein ALT, alanine aminotransferase; AST, aspartate transaminase.

* , *p* is significant at the level of < 0.05. (*, Student’s *t*; **, Mann–Whitney *U*).

**TABLE 3:** Comparison of laboratory and clinical parameters before and after lopinavir/ritonavir treatment in non-surviving patients.

Variables (*N* = 27)	Before treatment x̄ ± SD	After treatment x̄ ± SD	*p* *
Lymphocyte (K/mm^3^)	1.12 ± 0.66	0.87 ± 0.46	0.09*
Neutrophil (K/mm^3^)	7.8 ± 4.32	10.78 ± 7.49	0.07*
WBC (K/mm^3^)	10.57 ± 7.5	32.82 ± 100.39	0.26**
Platelet (K/mm^3^)	217.4 ± 123	250.9 ± 134.94	0.14*
CRP (mg/L)	136.97 ± 166.44	177.45 ± 71.14	0.001**
LDH (U/L)	859.92 ± 472.1	1439.24 ± 2029.4	0.06**
D-dimer(µg/L)	4717.55 ± 8958.7	6827.74 ± 8819.9	0.29**
Ferritin (mg/L)	1129.46 ± 1887.28	1309.35 ± 1311.79	0.21**
ALT (IU/L)	35.32 ± 25.53	123.14 ± 274.49	0.009**
AST (IU/L)	42.42 ± 26.31	148.13 ± 367.31	0.04**
Total bilirubin (mg/dL)	0.85 ± 0.63	3.53 ± 8.39	0.14*
Fibrinogen (µmol/L)	655.8 ± 150.65	586.5 ± 203.57	0.23*
SpO_2_ (%)	88.48 ± 6.61	74.26 ± 3.44	< 0.0001*

*N*, number; SD, standard deviation; WBC, white blood cell; CRP, C-reactive protein; LDH,lactate dehydrogenase; ALT, alanine aminotransferase; AST, aspartate transaminase; SpO2, oxygen saturation in room air.

* , *p* is significant at the level of < 0.05. (*, Paired t; **, Wilcoxon)

**TABLE 4:** Comparison of laboratory and clinical parameters before and after lopinavir/ritonavir treatment in surviving patients.

Variables (*N* = 46)	Before treatment x̄ ± SD	After treatment x̄ ± SD	*p* *
Lymphocyte (K/mm^3^)	1.23 ± 0.57	1.38 ± 0.55	0.16*
Neutrophil (K/mm^3^)	4.83 ± 3.09	4.38 ± 2.84	0.20*
WBC (K/mm^3^)	21.74 ± 73.19	13.31 ± 4.12	0.68**
Platelet (K/mm^3^)	187.44 ± 73.34	318.1 ± 141.63	< 0.0001*
CRP (mg/L)	52.11 ± 58.54	93.99 ± 156.03	0.21**
LDH (U/L)	511.61 ± 271.7	410.56 ± 100.3	0.02*
D-dimer (µg/L)	819.3 ± 937.83	947.5 ± 940.19	0.74**
Ferritin (mg/L)	480.49 ± 520.37	444.56 ± 355.79	0.33**
ALT (IU/L)	30.25 ± 13.46	47.27 ± 38.96	0.002*
AST (IU/L)	28.09 ± 14.17	29.98 ± 21.53	0.53*
Total bilirubin (mg/dL)	0.59 ± 0.31	2.29 ± 9.2	0.24*
Fibrinogen (µmol/L)	641.08 ± 161.31	672.67 ± 209.05	0.63*
SpO_2_ (%)	92.2 ± 6.94	94.83 ± 5.28	0.02*

*N*, number; SD, standard deviation; WBC, white blood cell; CRP, C-reactive protein; LDH, lactate dehydrogenase; ALT, alanine aminotransferase; AST, aspartate transaminase; SpO_2_, oxygen saturation in room air.

* , *p* is significant at the level of < 0.05 (*, Paired *t*; **, Wilcoxon).

Age (OR 1.09, 95% confidence interval [95% CI] 1.03–1.14), smoking (OR 6.18, 95% CI 1.7–22.42), presence of comorbid disease (OR 31.73, 6.26–153.56), coronary artery disease (CAD) (OR 9.26, 95% CI 1.79–47.77), arrhythmia (OR 13.8, 95% CI 1.56–122.22), hypertension (OR 14, 95% CI 4.28–45.74), diabetes (OR 7.22, 95% CI 2–25.99) and CHF (OR 10.22, 95% CI 1.13–92.93) were statistically associated with increased mortality (*p* < 0.05; Table 5). Also increased neutrophil (OR 1.26, 95% CI 1.08–1.46), CRP (OR 1.01, 95% CI 1.003–0.02), LDH (OR 1.002, 95% CI 1.001–004), D-dimer (OR 1.00, 95% CI 1.002–1.003) and AST levels (OR 1.05, 95% CI 1.02–1.08 were associated with increased mortality [Table 5]).

**TABLE 5:** Factors related with mortality.

Variables	Odds ratio	Lower (% 95 CI)	Upper (% 95 CI)	*p* *
Age	1.09	1.03	1.14	< 0.05
Smoking	6.18	1.70	22.42	< 0.05
Comorbidities	31.73	6.56	153.56	< 0.05
CAD	9.26	1.79	47.77	< 0.05
Arrhythmia	13.80	1.56	122.22	< 0.05
Hypertension	14.00	4.28	45.74	< 0.05
Diabetes	7.22	2.00	25.99	< 0.05
CHF	10.22	1.13	92.93	< 0.05
CRP	1.01	1.003	0.02	< 0.05
LDH	1.002	1.001	1.004	< 0.05
D-dimer	1.001	1.002	1.003	< 0.05
Neutrophil	1.26	1.08	1.46	< 0.05
AST	1.05	1.02	1.08	< 0.05

CAD, coronary artery disease; CHF, congestive heart failure; CRP, C-reactive protein; LDH, lactate dehydrogenase; AST, aspartate transaminase.

* , *p* is significant at the level of < 0.05 (Odds ratio).

## Discussion

In this retrospective study, we reviewed the patients who died and survived under Lpv/r treatment in severe and critical COVID-19 patients in terms of demographic characteristics, smoking, comorbidities, laboratory parameters and clinical outcomes. A total of 27 (36.9%) of the 73 patients died while under treatment. According to the study conducted by Cao et al.^9^ in patients with severe COVID-19 pneumonia, the mortality rate was quite high depending on the severity of the disease in the population received (36.9% vs. 22.1%).

Advanced age, active smoking, comorbidity (especially hypertension, diabetes, arrhythmia, CAD) were found to be associated with mortality. Similar to our study, Zhou et al.^12^ showed that mortality increased in the presence of advanced age and comorbidity especially hypertension, diabetes and CAD (*p* < 0.05) was associated with mortality in patients with COVID-19, but not smoking (*p* = 0, 21). In a recent systematic review, smoking has been shown to increase the progression of the disease in COVID-19 patients, and the symptoms were more severe in these patients, but the effect on mortality was controversial.^13^

Computed tomography findings in COVID-19 pneumonia range from normal appearance to diffuse lung involvement.^14^ In our study, the radiological distribution was classified from light to heavy (stage 1–4). The majority of patients (87.7%) were patients with moderate and advanced (stage 2–4) involvement. Mortality was not observed in patients with mild involvement (stage 1). Although the radiological prevalence and disease severity are thought to be parallel, there are no studies in the literature showing the relationship between radiological prevalence and mortality.

The early use of Lpv/r in SARS patients has been shown to significantly reduce mortality (15.6% and 2.3%, respectively) and PCR negation time (28.8% vs. 2.4%, respectively). Lopinavir/ritonavir early onset in SARS patients has been suggested to be used as a treatment.^5,6^ The positive effect of using Lpv/r as an initial treatment in SARS patients has not yet been demonstrated in COVID-19 patients.^9^ In our study, Lpv/r treatment was initiated in the survivor group on an average of 3.9 ± 2.4 days, and in the non-survivor group on an average of 3.7 ± 3.1 days (*p* = 53). There was no difference between symptom duration until hospitalisation, total length of stay and COVID-PCR negativity time between the two groups (*p* > 0.05).

In addition to routine tests in the determination of progression and fatal complications in COVID-19 patients, it is recommended to use CRP because it is parallel with LDH, D-dimer, ALT, AST and IL6.^15,16^ Ponti et al.^17^ stated that high AST, ALT, total bilirubin, LDH, D-dimer, CRP, WBC and low lymphocyte values were associated with progression. Similar to our study, Chen et al.^18^ compared survivor (*n* = 113) and non-survivor (*n* = 161) COVID 19 patients in their study, non-survivor group ALT, AST, LDH, D-dimer, cardiac troponins and creatinine levels were significantly higher. In our study, pre-treatment neutrophil, LDH, D-dimer, CRP, AST and total bilirubin levels were significantly higher in the non-survivor group (*p* < 0.05). When we compared laboratory values before and after the treatment, we saw that the survivor group showed an increase in platelet, CRP, ALT and SpO_2_ and a statistically significant decrease in LDH (*p* < 0.05). In non-survivor group, AST, ALT, CRP increased and SpO_2_ decreased (*p* < 0.05). Also increased neutrophil (OR 1.26, 95% CI 1.08–1.46), CRP (OR 1.01, 95% CI 1.003–0.02), LDH, (OR 1.002, 95% CI 1.001–004), D-dimer (OR 1.00, 95% CI 1.002–1.003) and AST levels (OR 1.05, 95% CI 1.02–1.08) were associated with increased mortality.

Publications on the use of Lpv/r in COVID-19 patients are very limited. Cao et al.^9^ found no significant difference between Lpv/r and standard treatment in terms of clinical improvement (hazard ratio for clinical improvement, 1.31; 95% confidence interval [CI], 0.95–1.80) and mortality (9.2% vs. 25.0%, respectively, difference, –5.8 percentage points; 95% CI, –17.3–5.7, respectively) in their study comparing standard treatment with Lpv/r in 199 patients with severe COVID-19. A combination of lopinavir/ritonavir/umifenovir was demonstrated to provide improvement of symptoms of four patients with COVID-19.^7^ Deng et al.^8^ in their study compared Lpv/r treatment alone with the combination of umifenovir + Lpv/r in patients with COVID-19 who did not receive invasive respiratory support, nasopharyngeal swab negation on day 7 (75% and 35%) and radiological recovery was significantly different in favour of the combination group. İn another retrospective study comparing Lpv/r and umifenovir alone, no difference was found between the groups in terms of improvement of the symptoms and decreasing viral load.^19^

## Limitations

This study is a unique example of a limited number of studies conducted on patients with COVID-19 pneumonia who were treated with Lpv/r. The most important limitation of the study was its retrospective nature. Therefore, adverse effects because of Lpv/r use could not be evaluated in this study. However, no patient had to discontinue the drug because of side effects. In 27.4% (*n* = 20) of the patients, COVID-19 PCR was negative. Chest CT, clinical and laboratory findings in all of these patients were consistent with COVID-19. All patients included in the study were patients with severe COVID-19. Lopinavir/ritonavir treatments were used in accordance with the Scientific Committee of Turkish Ministry of Health guidelines. As all patients with severe COVID-19 were treated with Lpv/r, we could not include a control group and could not make a comparison between the patient and control groups in terms of Lpv/r.

## Conclusion

Advanced age, active smoking, comorbidity, especially hypertension, diabetes, arrhythmia, CAD and neutrophil, CRP, LDH, D-dimer and AST levels were associated with mortality. The survivor group showed an increase in platelet, CRP, ALT and SpO_2_ and a statistically significant decrease in LDH. As AST, ALT and CRP were increased and SpO_2_ was decreased, LPV/r appeared to have worsened biochemical parameters in the non-survivor group. The efficacy of LPV/r should be evaluated with clinical randomised trials.
